# Trends in prevalence of multi drug resistant tuberculosis in sub-Saharan Africa: A systematic review and meta-analysis

**DOI:** 10.1371/journal.pone.0185105

**Published:** 2017-09-25

**Authors:** Baba Maiyaki Musa, Aishatu L. Adamu, Najibah A. Galadanci, Bashir Zubayr, Chisom N. Odoh, Muktar H. Aliyu

**Affiliations:** 1 Department of Medicine, Bayero University, Kano, Nigeria; 2 Department of Medicine, Aminu Kano Teaching Hospital, Kano, Nigeria; 3 Department of Community Medicine, Bayero University, Kano, Nigeria; 4 Department of Community Medicine, Aminu Kano Teaching Hospital, Kano, Nigeria; 5 Department of Hematology, Bayero University, Kano, Nigeria; 6 Department of Hematology, Aminu Kano Teaching Hospital, Kano, Nigeria; 7 IHVN Regional Office, Institute of Human Virology, Kano, Nigeria; 8 University of Louisville, Louisville, Kentucky, United States of America; 9 Vanderbilt Institute for Global Health, Vanderbilt University School of Medicine, Nashville, Tennessee, United States of America; 10 Department of Health Policy, Vanderbilt University School of Medicine, Nashville, Tennessee, United States of America; The University of Hong Kong, CHINA

## Abstract

**Background:**

Multidrug resistant tuberculosis (MDR-TB), is an emerging public health problem in sub-Saharan Africa (SSA). This study aims to determine the trends in prevalence of MDR-TB among new TB cases in sub-Saharan Africa over two decades.

**Methods:**

We searched electronic data bases and accessed all prevalence studies of MDR-TB within SSA between 2007 and 2017. We determined pooled prevalence estimates using random effects models and determined trends using meta-regression.

**Results:**

Results: We identified 915 studies satisfying inclusion criteria. Cumulatively, studies reported on MDR-TB culture of 34,652 persons. The pooled prevalence of MDR-TB in new cases was 2.1% (95% CI; 1.7–2.5%). There was a non-significant decline in prevalence by 0.12% per year.

**Conclusion:**

We found a low prevalence estimate of MDR-TB, and a slight temporal decline over the study period. There is a need for continuous MDR-TB surveillance among patients with TB.

## Introduction

Multi drug resistant tuberculosis (MDR-TB) is a global public health challenge, especially in sub-Saharan Africa (SSA), where it adds to the burden of other communicable and non-communicable diseases bedeviling the region. In 2015 WHO estimated 480,000 new cases of MDR-TB worldwide. An additional 100,000 cases were rifampicin-resistant TB (RR-TB), making them eligible for MDR-TB treatment.[1] Cases of MDR-TB continue to rise—whereas, in 2014, MDR-TB constituted 3.3% of new Tuberculosis (TB) cases, in 2015 an estimated 3.9% of new and 21% of previously treated TB cases were found to be (MDR/RR-TB). [2]

The emergence of MDR-TB is linked to weak TB control programs and sub-optimal TB case management.[3]This realization is more pronounced in SSA, due to its limited resources and dearth of trained TB health work force[4]. Indeed, by 2016 only 18 out of the 46 SSA countries reported ever conducting a national MDR-TB survey,[1] a major constraint to proper planning and to achieving the End TB Strategy targets.[5, 6]

In view of the paucity of national MDR-TB survey in SSA a number of studies have attempted to estimate the burden of MDR-TB in SSA. Lukoye in The Netherlands, [7] found a pooled MDR-TB prevalence estimate of 1.5% (95% CI 1.0–2.3), while Berhan in Ethiopia, reported a five-fold increased risk of MDR-TB among previously treated tuberculosis (TB) cases compared to new cases.[8] Available literature on prevalence of MDR-TB in SSA depicts remarkable within and between country differences.[7, 8]

Considering the significant within and between country variability in reported MDR-TB prevalence in SSA, this review aims to provide a comprehensive and up-to-date evaluation of the burden and trend of the MDR-TB among new cases in SSA. Findings from this work can inform intervention strategies and future MDR-TB monitoring.

## Materials and methods

### Data search

Three of the authors independently searched for literature related to prevalence of MDR-TB in selected bibliographic databases, namely PubMed/Medline, Institute of Science Information (ISI), Google Scholar, African Journals Online (AJOL), BIOLINE Cochrane, HINARI, Embase, and Scopus. The search was limited to 1st January 1997 to 31st May 2017, with no language restriction. The reference list of retrieved papers was further searched to identify additional relevant literature. We contacted authors for unpublished literature and publications with inconclusive reporting. Subject specialists were further contacted for unpublished write ups.

The search strategy combined keywords and medical subject headings (MeSH). The following key search terms were used: “prevalence” or “burden”; “Mycobacterium tuberculosis” or “tuberculosis”; “anti-TB drug susceptibility” or “anti-TB drug resistance”; “Resistant TB” or “MDRTB” and “Africa” or “Sub-Saharan Africa”. These string were further attached to names of SSA countries. Details of the Pubmed search strategy (one of the databases) are depicted in S1 Table.

### Inclusion and exclusion criteria

Data on MDR in new TB cases from studies reporting prevalence of MDR-TB among new TB cases and data on MDR TB in new cases from studies with disaggregated data on new and retreatment cases were included in the final analysis. We used adjudication to resolve discrepancies on articles to be included.

We excluded from the analysis case reports case series, studies without primary data, commentaries, literature reviews, studies adjudged to have poor quality based on NIH criteria,[9] studies presenting data before 1997, studies with data unrelated to MDR-TB, studies without adequate data, studies not reporting the culture or DST method, studies not reporting the sampling method, studies reporting only data on retreatment cases, and studies reporting new and retreatment cases without disaggregation.

### Data extraction

The following information were extracted from the eligible studies: first author’s name; year of publication; country of study; study design; study description; study setting; culture and DST methods; numeric count of MDR-TB cases; numeric count of new TB cases screened; age group; HIV prevalence in the study (HIV prevalence at national level for each country of interest was collected from the UNAIDS Report 2017); and type of resistance tested. We coded data based on authors name, study country, and year of study. Multiple Coder agreement was assessed using Cohen’s kappa.

### Operational definitions

WHO defines resistance among new cases as resistance to one or more anti-tuberculosis drugs in patients that have never been treated for TB, while “MDR-TB” implies that TB is resistant to at least rifampicin and isoniazid. This is to distinguish it from MDR-TB in old cases which would refer to occurrence of MDR in patients with prior TB treatment. We defined SSA sub-regions as follows: Eastern sub-region; West Africa sub-region; Southern sub-region and Central Africa sub-region. Identification of TB was done using Löwenstein-Jensen BACTEC, and GeneXpert MTB/RIF assay (Xpert^®^ MTB/RIF assay (Cepheid, Sunnyvale, CA, USA). The drug sensitivity test (DST) methods used were the proportion method, the absolute concentration method GeneXpert MTB/RIF assay, and BACTEC method. While some studies used LJ, BACTEC MGIT 960 system, or BACTEC 460 TB radiometric as a standalone test, others used a mix of diagnostic methods.

### Quality of included studies

Two of the authors independently assessed quality of included studies using the NIH Quality Assessment Tool for Observational Cohort and Cross-Sectional Studies.[9] Studies were assessed with questions appropriate to their study design. We graded quality as good (G) if rating was at least 70%, fair (F) if at least 50%, and poor (P) if less than 50%.

### Statistical analyses

The primary outcome measure was prevalence of MDR-TB. Standard error of prevalence was estimated using binomial probability distribution. Overall and sub-group pooled effect size was estimated using random effects model with DerSimonian-Laird method by calculating the pooled prevalence estimate and confidence interval, based on the weighted least square (weighting was given by the reciprocal sum of between and within study variances). Between-studies heterogeneity was evaluated using Cochran’s Q test. A priori, we defined low, medium and high heterogeneity as a Cochran’s Q of 25%, 50%, and 75%, respectively. To determine potential confounders and prevalence trend, meta-regression analysis was done. We appraised publication bias by funnel plot, Begg’s rank correlation methods and Egger’s weighted regression test. Prevalence derived from included studies were depicted on funnel plot by diamond points, while augmented data prevalence obtained using the trim and fill method are represented by a diamond symbol within a square. All analyses were performed using STATA software (version 11). We adopted 0.05 as significant level for the Cochran’s test. We ran a sensitivity analysis to assess the effect of sample size on the aggregate prevalence. The null hypothesis of this study assumes that the all the studies have the same prevalence in the various populations studied.

## Results

### Characteristics of included studies

**Fig 1:**
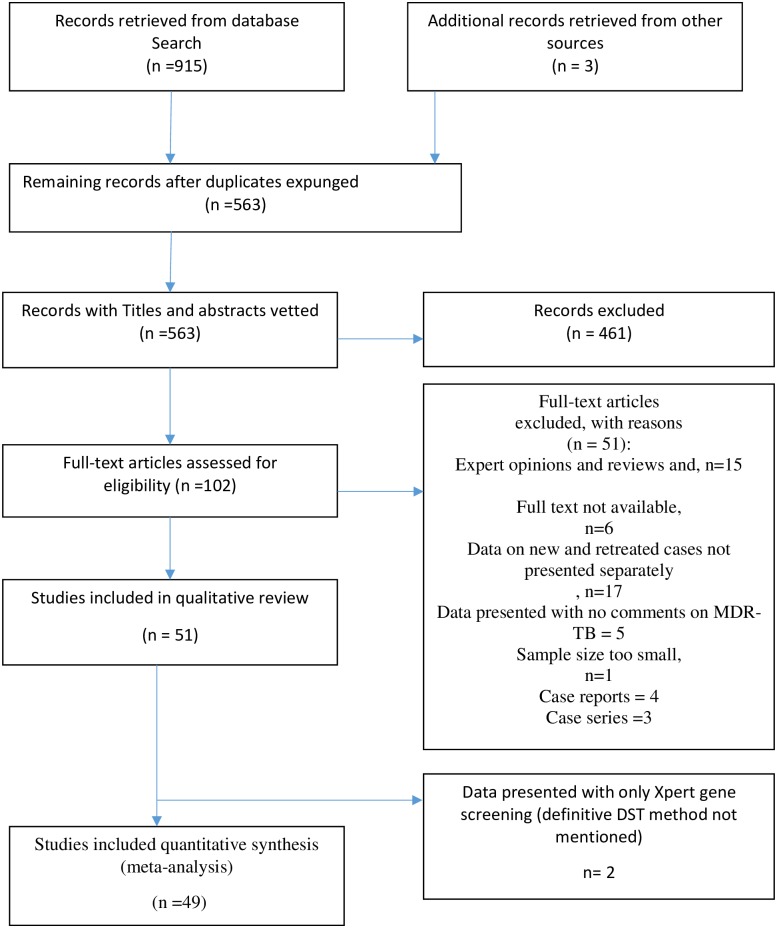
PRISMA flow diagram showing study screening and selection procedure.

A total of 915 citations were found, out of which 51 articles were included in the qualitative review.[10–60], and 49 articles that met the inclusion criteria were included in the meta-analysis. (two were finally excluded on account of using Gene Xpert alone for diagnosing MDR TB),Fig 1. These comprised of 35 cross-sectional studies, ten prospective cohort studies, and four retrospective cohort studies. Characteristics of included studies are summarized in (S3 Table). We used PRISMA and MOOSE checklist for assessment of meta-analysis guideline compliance(S4 and S5 Tables).

### Quality assessment

Based on the 10 quality domains assessed, most of the studies satisfied at least six of the quality benchmarks. The most common quality benchmark failed by the studies were: inadequate sample size, sample size justification, power description, or variance and effect estimates provided. While all included studies had a score of at least 50%, 32(65%) had a score of at least 70% Details of grading procedure is presented in S2 Table.

### Pooled prevalence of MDR TB among new TB patients

**Fig 2:**
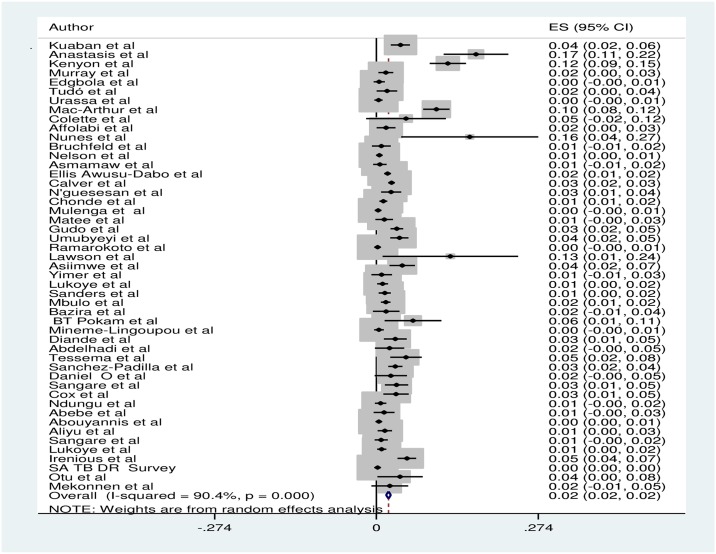
Forest plot showing pooled and individual weighted prevalence of included studies. ES: effect size, CI: confidence interval.

The meta-analysis derived a pooled MDR-TB prevalence of 2.0% (95% CI; 1.7–2.4%) for new cases of TB in SSA. [Fig 2] The identified number of new MDR-TB cases was based on culture of samples from 34,056 individuals, spanning 20 years (1997–2017).

Table 1 shows the subgroup analyses for the prevalence of MDR-TB in new TB cases by study design, region, and type of survey and study year. We found a wide disparity in the prevalence of evaluated characteristics.

**Table 1:** Disaggregated prevalence of MDR-TB by study attributes and their measure of heterogeneity.

Category stratified by	Sub-group	Number of studies reviewed	Prevalence of MDR-TB % (95%CI)	Heterogeneity test	
				I^2^%	p value
Study design	Prospective	10	1.4(0.6–2.1)	82.2	0.001
	Retrospective	4	4.1(1.6–6.6)	89.3	0.001
	Cross-sectional	35	2.2(1.7–2.7)	88.4	0.001
Region	Western	12	1.9(1.2–2.6)	54.4	0.001
	Eastern	20	1.7(1.1–2.2)	87.2	0.001
	Central	8	2.1(1.1–3.0)	75.2	0.001
	Southern	9	3.1(2.1–4.2)	96.2	0.001
Type of survey	National survey	13	1.5(1.0–2.0)	93.3	0.001
	Sub-national survey	36	2.5(1.9–3.2)	86.0	0.001
Year of study	1997	2	10.2(2.3–22.7)	94.8	0.001
	1999	4	3.6(0.5–6.7)	94.0	0.001
	2001	2	5.2(4.4–14.8)	98.5	0.001
	2002	5	1.2(0.1–2.6)	61.1	0.001
	2004	2	1.3(0.1–2.6)	75.5	0.001
	2005	2	2.6(2.0–3.1)	0.0	0.001
	2006	3	0.8(0.1–1.5)	60.1	0.001
	2007	4	2.9(0.2–5.6)	94.6	0.001
	2008	7	1.7(0.9–2.5)	49.3	0.001
	2009	5	2.6(0.9–4.4)	82.8	0.001
	2010	7	1.5(0.6–2.3)	67.6	0.001
	2011	3	2.2(0.1–4.5)	91.8	0.001
	2012	2	1.6(2.0–5.2)	73.1	0.001
	2014	1	2.3(0.8–5.4)	0.0	0.001

**Fig 3:**
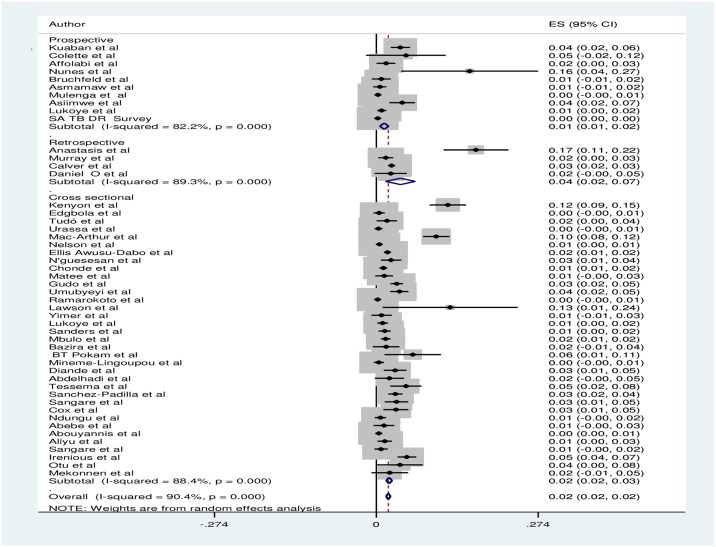
Forest plot showing pooled MDR TB prevalence in new TB cases stratified by study design of included studies. ES: effect size, CI: confidence interval.

The prevalence of MDR-TB was highest in retrospective studies with a prevalence of 4.1% (95% CI: 1.6–6.6) and lowest in prospective studies 1.4(95% CI: 0.6–2.10). [Fig 3] Pooled prevalence of MDR-TB in national surveys was 1.5% (95% CI:1.0–2.0) and 2.5% (95 CI: 1.9–3.2) in sub national surveys. When assessed by region, prevalence varied from 3.1% (95% CI: 2.1–4.2) in the Southern African region to1.7% (95% CI: 1.1–2.2) in Eastern Africa. Table 1

When sensitivity analysis was performed by study year the prevalence of MDR-TB ranged from 10.2% (95% CI: 2.3–22.7) in studies done in 1997, to 0.8% (95% CI: 0.1–1.5) in studies done in 2006. [Table 1] Studies done in 2008 and 2010; the years with highest number of reported studies (each seven) had a pooled prevalence of 1.7% (95% CI0.9–2.5) and 1.5% (95% CI: 0.6–2.3) respectively. [Table 1]

**Fig 4:**
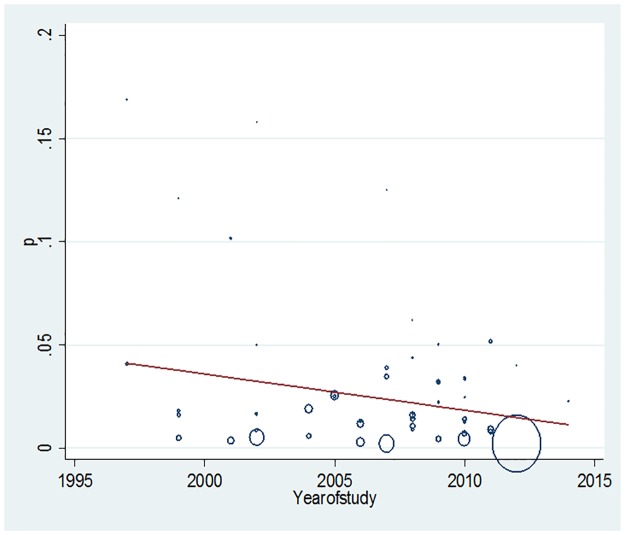
Meta-regression plot showing sub-Saharan African (S SA) trend in MDR-TB prevalence in new cases over two decades. P: MDR TB prevalence in new cases, circle size proportionate to size of studied population in each year.

We assessed evidence of trend in prevalence of MDR-TB in new TB cases using meta-regression. There was a non-significant decline in the prevalence of MDR-TB, at a rate of 0.18% per year (p value = 0.07). [Fig 4] Study year, region, population, age, or method of screening were not found to be significant confounders of MDR-TB prevalence estimates. Table 2

**Table 2:** Meta-regression: Association between pooled prevalence of new MDR TB cases and study characteristics.

CHARACTERISTICS	¶ Univariate coefficient 95% CI: ()	P value	Adjusted coefficient	P value
Year of study	-0.0018 (-.0037 .00015)	0.070		
Study design				
Cross sectional	Ref		-	
Prospective	-0.0056 (-0.026 0.016)	0.598	-	
Retrospective	0.016 (-0.0149 .0482)	0.296		
Region,			-	
Central Africa	Ref		-	
East Africa	-0.0023 (-0.026–0.022)	0.850	-	
Southern Africa	0.016 (-0.012–0.043)	0.259	-	
West Africa	.0019786 (-0.025–0.029)	0.882	-	
Population	Collinearity	-	-	
Age,	Collinearity	-	-	
Method of screening			-	
BACTEC 460	Ref		-	
BACTEC 960	.00233(-0.020–0.024)	0.836	-	
LJ	.00906 (-0.050 0.068)	0.762	-	
HIV prevalence in study	-0.0001 (-0.0006 .0005)	0.696	-	

^¶^The regression coefficients are the estimated increase in the MDR TB prevalence per unit increase in the study characteristics

**Fig 5:**
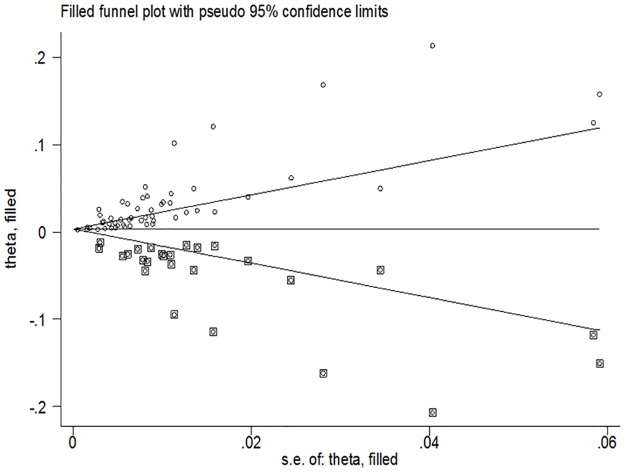
Trim and filled funnel plot depicted study derived and data augmented prevalence of MDR-TB in new cases for SSA. Theta: the effect (prevalence) estimate, SE: standard error; diamond shapes represent study derived prevalence; diamond within square represent data augmented prevalence.

We found evidence of publication bias using Egger’s and Begg’s test statistics (p value = 0.001 for both tests). The funnel plot demonstrated evidence of asymmetry. [Fig 5].

## Discussion

With a pooled prevalence of 2.1%, we have demonstrated a low prevalence of MDR-TB in new cases of TB in SSA. We have also shown evidence of a declining trend in the prevalence of MDR-TB in new TB cases over a period of 20 years.). If it is assumed that Africa had a TB incidence of 2,720/100,000 population in 2015,[1] then we would be projecting an MDR-TB burden in new cases of 54/100,000 population, with uncertainty interval of (47–62)/100,000 population. With a review spanning 2007–2017, to our knowledge this study is the most recent extensive review of the burden of MDR-TB in new cases in SSA. It is also the first study, to our knowledge, to assess the trend in the prevalence of MDR-TB in SSA using meta-analytical methods.

Our findings are consistent with WHO estimates of MDR-TB in new cases in Africa of 1.9%, (95% CI 0.6–3.3).[3] Similarly, an earlier meta-analysis (2013) reported a pooled prevalence of 1.5% (95% CI 1.0–2.3).[7] A recent MDR-TB survey in three SSA countries also demonstrated a similar low prevalence of MDR-TB in new cases (prevalence range: 0.6–2.0).[61]

Whereas, we found a non-significant declining trend in the prevalence of MDR-TB in new cases, Xiao-chun and Mukinda found rising trend in China and South Africa respectively[62, 63] However, our study modelled a composite measure of MDR-TB from different countries, whereas, these studies assessed single country trends. These differences between our findings and others buttress the need for continuous MDR-TB surveillance as a necessary tool for effective TB control. Drug resistance surveillance allows tracking of ongoing resistance transmission, and creates an enabling environment for planning.[3] Continuous surveillance of MDR-TB would not only assess epidemiological trends, but also allow for timely determination and response to MDR-TB outbreaks. Further, continuous surveillance helps to track the effectiveness of national and global TB prevention and control interventions, and allows for optimum forecasting of patient treatment needs.[64]

We noted disparity in pooled prevalence based on study design. The highest prevalence levels were observed in retrospective cohort studies, while the lowest prevalence values were observed in prospective studies. Similarly, pooled prevalence levels of national surveys were lower than values reported from sub-national surveys. Almost all national surveys included in the meta-analysis had cross- sectional study design, with their pooled prevalence being closer to the overall pooled prevalence. This finding may reflect the good rigor involved in designing national surveys.

The pooled prevalence of MDR-TB in new cases was highest in studies from Southern Africa and lowest in Eastern African studies, while the pooled prevalence for West and Central African sub-regions were mid-way. Although there is high heterogeneity within and between Southern African countries, it is not surprising to find relatively high primary MDR-TB resistance there, because of the substantial contribution of South Africa to the global and regional burden of TB. Our findings are consistent with an earlier WHO report of relatively high prevalence of MDR-TB in new cases in Southern Africa.[3] Southern Africa has one of the highest regional global prevalence in HIV. Although conclusive evidence of the association between HIV and risk of developing MDR-TB is lacking, it is plausible that high HIV burden could be contributing to the observed MDR-TB burden in this sub-region.[7, 8]

Although the pooled prevalence trend obtained in this study using regression modeling may suggest a declining trend in MDR-TB, sensitivity analysis on year of study was heterogeneous, with no clear discernable pattern. This finding could be explained by the disparate study design, varying study population sizes, and settings in which these studies were conducted.

Many SSA countries have adopted WHO recommendation of using Xpert MTB/RIF assay in screening for MDR-TB in new cases. However its utility when used as a standalone might be limited, considering its relatively low positive predictive value in populations with low burden of MDR-TB. In view of the cost to individuals and health system of unnecessarily treating MDR-TB in SSA, we advocate augmenting Xpert MTB/RIF assay with drug sensitivity testing DST.[65]

Our finding of significant publication bias may be a reflection of wide heterogeneity in reported prevalence of MDR-TB and of potential gaps in data from unreported prevalence in certain sub populations. We have attempted to account for missing data points using the fitted data from iteration based on the “trim and fit methods” of assessing publication bias. Other possible reasons for this finding include: differences in study sample sizes, TB culture methods, and variation in study rigor. Our finding may also reflect temporal variability in prevailing risk for MDR-TB among study populations. We have tried to include only high quality with rating of at least 50%.[9]

Our study had some of limitations. Not all countries in SSA had reported MDR-TB national surveys, as such we had to rely on prevalence assessment in sub-populations. Nevertheless, these were considered fairly representative. We equally had to exclude some studies because they did not clearly delineate data into new or relapse cases.

### Conclusion

This study shows a low MDR-TB prevalence in new cases, and declining prevalence of MDR-TB over the two decades covered by this study. Perhaps this finding may not necessarily suggest a low MDR-TB prevalence and prevalence decline, but rather limitation in the number of reports of national MDR-TB surveys in SSA. This study depicts gaps in documentation of MDR-TB cases in several SSA countries. In countries with available data, documentation is often limited to sub populations, without the necessary national representativeness of a national survey. While data from national MDR-TB surveys are rewarding as interim measures, a truly robust understanding would be better achieved if MDR-TB assessment is integrated into routine TB care to achieve continuous drug resistance surveillance. A better understanding of epidemiological trends in drug resistance at the global and national levels can be achieved through repeated surveys and, ultimately, by establishing proper MDR-TB surveillance systems. We advocate for rigorous and representative MDR-TB surveillance systems that would generate data to guide the development and prioritization of MDR-TB control policies within and across national borders.

## Supporting information

S1 Table Search strategy used for one of the databases (PubMed). MeSH: medical sub-heading.
(DOCX)

S2 Table Details of quality rating for included studies. Yes: satisfies criteria, No doesn’t satisfy criteria, G: good score (≥ 70%), F: fair score (≥50%).
(DOCX)

S3 Table Details of included study characteristics. LJ: Löwenstein-Jensen, HAIN: Hain assay, Xpert: Xpert^®^ MTB/RIF assay, MIGT: Mycobacterial growth indicator tube.
(DOCX)

S4 Table PRISMA checklist of the systematic review. (DOC)

S5 Table MOOSE checklist for observational studies. (DOC)
