# Supplementary material for: Trends in prevalence of multi drug resistant tuberculosis in sub-Saharan Africa: A systematic review and meta-analysis
Source: PLoS One. 2017 Sep 25;12(9):e0185105. doi: 10.1371/journal.pone.0185105 (PMC5612652; doi:10.1371/journal.pone.0185105)
Supplement: S1 Table — MeSH: medical sub-heading. (DOCX) [file pone.0185105.s001.docx]

**S1 Table. Search strategy used for one of the databases (Pubmed)**

| Pubmed | | | |
| --- | --- | --- | --- |
| Group | | Search terms |  |
|  |  | MeSH (subterms in MeSH) | *Non MeSH* |
| #1 |  | “prevalence”  or  “burden” |  |
| #2 |  | “Mycobacterium tuberculosis” or  “tuberculosis” |  |
| #3 |  | “anti-TB drug susceptibility”  Or  “anti-TB drug resistance” | Anti-tuberculosis resistance  Rifampicin-resistant tuberculosis  Isoniazid-resistant tuberculosis  Drug-resistant tuberculosis  Multidrug-resistant tuberculosis |
| #4 |  | “Resistant TB”  Or  “MDR TB” |  |
| #5 |  | “Africa” or “Sub-Saharan Africa” | List of SSA countries |
|  | Limit | 1st January 1997  to  31st May 2017 |  |
| Strategy | #1 AND #2 AND #3 AND #4 AND #5 | | |
