# Supplementary material for: Trends in prevalence of multi drug resistant tuberculosis in sub-Saharan Africa: A systematic review and meta-analysis
Source: PLoS One. 2017 Sep 25;12(9):e0185105. doi: 10.1371/journal.pone.0185105 (PMC5612652; doi:10.1371/journal.pone.0185105)
Supplement: S2 Table — Yes: satisfies criteria, No doesn’t satisfy criteria, G: good score (≥ 70%), F: fair score (≥50%). (DOCX) [file pone.0185105.s002.docx]

|  | **Author** | **Was the research question or objective in this paper clearly stated?** | **Was the study population clearly specified and defined?** | **Were all the subjects selected or recruited from the same or similar populations (including the same time period)?** | **Were inclusion and exclusion criteria for being in the study pre-specified and applied uniformly to all participants?** | **Was a sample size justification, power description, or variance and effect estimates provided?** | **Was the sample size adequate for the evaluation?** | **Were the outcome measures (dependent variables) clearly defined, valid, reliable, and implemented consistently across all study participants?** | **For the analyses in this paper, were the exposure(s) of interest measured prior to the outcome(s) being measured?** | **Was loss to follow-up after baseline 20% or less?** | **Were key potential confounding variables measured and adjusted statistically for their impact on the relationship between exposure(s) and outcome(s)** | **Quality grading** |
| --- | --- | --- | --- | --- | --- | --- | --- | --- | --- | --- | --- | --- |
| 1 | Kuaban et al | Yes | Yes | Yes | Yes | Yes | Yes | Yes | Yes | Yes | Yes | G |
| 2 | Anastasis et al | Yes | Yes | Yes | Yes | No | No | Yes | Yes | Yes | No | F |
| 3 | Kenyon et al | Yes | Yes | Yes | Yes | Yes | Yes | Yes | Yes | Yes | Yes | G |
| 4 | Murray et al | Yes | Yes | Yes | Yes | Yes | Yes | Yes | Yes | Yes | Yes | G |
| 5 | Edgbola et al | Yes | Yes | Yes | Yes | Yes | No | Yes | Yes | Yes | No | F |
| 6 | Tudó et al | Yes | Yes | Yes | Yes | Yes | No | Yes | Yes | Yes | Yes | F |
| 7 | Urassa et al | Yes | Yes | Yes | Yes | Yes | No | Yes | Yes | Yes | Yes | F |
| 8 | Mac-Arthur et al | Yes | Yes | Yes | Yes | Yes | Yes | Yes | Yes | Yes | Yes | G |
| 9 | Colette et al | Yes | Yes | Yes | Yes | No | No | Yes | Yes | Yes | No | F |
| 10 | Affolabi et al | Yes | Yes | Yes | Yes | Yes | Yes | Yes | Yes | Yes | Yes | G |
| 11 | Nunes et al | Yes | Yes | Yes | Yes | No | No | Yes | Yes | Yes | No | F |
| 12 | Bruchfeld et al | Yes | Yes | Yes | Yes | No | Yes | Yes | Yes | Yes | No | F |
| 13 | Nelson et al | Yes | Yes | Yes | Yes | Yes | Yes | Yes | Yes | Yes | Yes | G |
| 14 | Asmamaw et al | Yes | Yes | Yes | Yes | No | No | Yes | Yes | Yes | No | F |
| 15 | Ellis Awusu-Dabo et al | Yes | Yes | Yes | Yes | Yes | Yes | Yes | Yes | Yes | Yes | G |

|  | Author | **Was the research question or objective in this paper clearly stated?** | **Was the study population clearly specified and defined?** | **Were all the subjects selected or recruited from the same or similar populations (including the same time period)?** | **Were inclusion and exclusion criteria for being in the study pre-specified and applied uniformly to all participants?** | **Was a sample size justification, power description, or variance and effect estimates provided?** | **Was the sample size adequate for the evaluation?** | **Were the outcome measures (dependent variables) clearly defined, valid, reliable, and implemented consistently across all study participants?** | **For the analyses in this paper, were the exposure(s) of interest measured prior to the outcome(s) being measured?** | **Was loss to follow-up after baseline 20% or less?** | **Were key potential confounding variables measured and adjusted statistically for their impact on the relationship between exposure(s) and outcome(s)** | Quality grading |
| --- | --- | --- | --- | --- | --- | --- | --- | --- | --- | --- | --- | --- |
| 16 | Calver et al | Yes | Yes | Yes | Yes | Yes | Yes | Yes | Yes | Yes | Yes | G |
| 17 | N'guesesan et al | Yes | Yes | Yes | Yes | Yes | Yes | Yes | Yes | Yes | Yes | G |
| 18 | Chonde et al | Yes | Yes | Yes | Yes | Yes | Yes | Yes | Yes | Yes | Yes | G |
| 19 | Mulenga et al | Yes | Yes | Yes | Yes | Yes | Yes | Yes | Yes | Yes | Yes | G |
| 20 | Matee et al | Yes | Yes | Yes | Yes | Yes | Yes | Yes | Yes | Yes | No | G |
| 21 | Gudo et al | Yes | Yes | Yes | Yes | Yes | Yes | Yes | Yes | Yes | Yes | G |
| 22 | Umubyeyi et al | Yes | Yes | Yes | Yes | Yes | Yes | Yes | Yes | Yes | Yes | G |
| 23 | Ramarokoto et al | Yes | Yes | Yes | Yes | Yes | Yes | Yes | Yes | Yes | Yes | G |
| 24 | Lawson et al | Yes | Yes | Yes | Yes | No | No | Yes | Yes | Yes | No | F |
| 25 | Asiimwe et al | Yes | Yes | Yes | Yes | Yes | Yes | Yes | Yes | Yes | Yes | G |
| 26 | Yimer et al | Yes | Yes | Yes | Yes | No | No | Yes | Yes | Yes | No | F |
| 27 | Lukoye et al | Yes | Yes | Yes | Yes | Yes | Yes | Yes | Yes | Yes | Yes | G |
| 28 | Sanders et al | Yes | Yes | Yes | Yes | Yes | Yes | Yes | Yes | Yes | Yes | G |

|  | Author | **Was the research question or objective in this paper clearly stated?** | **Was the study population clearly specified and defined?** | **Were all the subjects selected or recruited from the same or similar populations (including the same time period)?** | **Were inclusion and exclusion criteria for being in the study pre-specified and applied uniformly to all participants?** | **Was a sample size justification, power description, or variance and effect estimates provided?** | **Was the sample size adequate for the evaluation?** | **Were the outcome measures (dependent variables) clearly defined, valid, reliable, and implemented consistently across all study participants?** | **For the analyses in this paper, were the exposure(s) of interest measured prior to the outcome(s) being measured?** | **Was loss to follow-up after baseline 20% or less?** | **Were key potential confounding variables measured and adjusted statistically for their impact on the relationship between exposure(s) and outcome(s)** | Quality grading |
| --- | --- | --- | --- | --- | --- | --- | --- | --- | --- | --- | --- | --- |
| 29 | Mbulo et al | Yes | Yes | Yes | Yes | Yes | Yes | Yes | Yes | Yes | Yes | G |
| 30 | Bazira et al | Yes | Yes | Yes | Yes | No | No | Yes | Yes | Yes | No | F |
| 31 | BT Pokam et al | Yes | Yes | Yes | Yes | No | No | Yes | Yes | Yes | No | F |
| 32 | Mineme-Lingoupou et al | Yes | Yes | Yes | Yes | Yes | Yes | Yes | Yes | Yes | No | G |
| 33 | Diande et al | Yes | Yes | Yes | Yes | Yes | Yes | Yes | Yes | Yes | Yes | G |
| 34 | Abdelhadi et al | Yes | Yes | Yes | Yes | No | No | Yes | Yes | Yes | No | F |
| 35 | Tessema et al | Yes | Yes | Yes | Yes | Yes | Yes | Yes | Yes | Yes | No | G |
| 36 | Sanchez-Padilla et al | Yes | Yes | Yes | Yes | Yes | Yes | Yes | Yes | Yes | Yes | G |
| 37 | Daniel O et al | Yes | Yes | Yes | Yes | No | No | Yes | Yes | Yes | No | F |
| 38 | Sangare et al | Yes | Yes | Yes | Yes | Yes | Yes | Yes | Yes | Yes | Yes | G |
| 39 | Cox et al | Yes | Yes | Yes | Yes | Yes | Yes | Yes | Yes | Yes | No | G |
| 40 | Ndungu et al | Yes | Yes | Yes | Yes | Yes | Yes | Yes | Yes | Yes | No | G |
| 41 | Abebe et al | Yes | Yes | Yes | Yes | No | No | Yes | Yes | Yes | Yes | F |
| 42 | Abouyannis et al | Yes | Yes | Yes | Yes | Yes | Yes | Yes | Yes | Yes | Yes | G |

|  | Author | **Was the research question or objective in this paper clearly stated?** | **Was the study population clearly specified and defined?** | **Were all the subjects selected or recruited from the same or similar populations (including the same time period)?** | **Were inclusion and exclusion criteria for being in the study pre-specified and applied uniformly to all participants?** | **Was a sample size justification, power description, or variance and effect estimates provided?** | **Was the sample size adequate for the evaluation?** | **Were the outcome measures (dependent variables) clearly defined, valid, reliable, and implemented consistently across all study participants?** | **For the analyses in this paper, were the exposure(s) of interest measured prior to the outcome(s) being measured?** | **Was loss to follow-up after baseline 20% or less?** | **Were key potential confounding variables measured and adjusted statistically for their impact on the relationship between exposure(s) and outcome(s)** | Quality grading |
| --- | --- | --- | --- | --- | --- | --- | --- | --- | --- | --- | --- | --- |
| 43 | Aliyu et al | Yes | Yes | Yes | Yes | Yes | Yes | Yes | Yes | Yes | Yes | G |
| 44 | Sangare et al | Yes | Yes | Yes | Yes | Yes | Yes | Yes | Yes | Yes | No | G |
| 45 | Lukoye et al | Yes | Yes | Yes | Yes | Yes | Yes | Yes | Yes | Yes | Yes | G |
| 46 | Irenious et al | Yes | Yes | Yes | Yes | Yes | Yes | Yes | Yes | Yes | Yes | G |
| 47 | Halilu et al | Yes | Yes | Yes | Yes | No | No | Yes | Yes | Yes | No | F |
| 48 | South African Tuberculosis Drug Resistance Survey | Yes | Yes | Yes | Yes | Yes | Yes | Yes | Yes | Yes | Yes | G |
| 49 | Otu et al | Yes | Yes | Yes | Yes | Yes | Yes | Yes | Yes | Yes | No | G |
| 50 | Mekonnen et al | Yes | Yes | Yes | Yes | No | No | Yes | Yes | Yes | No | F |
| 51 | Okorie et al | Yes | Yes | Yes | Yes | Yes | Yes | Yes | Yes | Yes | Yes | G |

G: Good F: fair P: Poor

Graded quality, good (G) if rating was at least 70%, fair (F) if at least 50%, and poor (P) if less than 50%.
