# Supplementary material for: Trends in prevalence of multi drug resistant tuberculosis in sub-Saharan Africa: A systematic review and meta-analysis
Source: PLoS One. 2017 Sep 25;12(9):e0185105. doi: 10.1371/journal.pone.0185105 (PMC5612652; doi:10.1371/journal.pone.0185105)
Supplement: S3 Table — LJ: Löwenstein-Jensen, HAIN: Hain assay, Xpert: Xpert® MTB/RIF assay, MIGT: Mycobacterial growth indicator tube. (DOCX) [file pone.0185105.s003.docx]

|  | **Author** | **Country** | **Region** | **Year of study** | **Study design** | **Evaluation** | **Culture** | **Positive MDRTB** | **Number of new Tuberculosis cases** | **Quality grading** |
| --- | --- | --- | --- | --- | --- | --- | --- | --- | --- | --- |
| 1 | Kuaban et al | Cameroon | Southern Africa | 1997 | Prospective | Sub national survey | LJ | 23 | 566 | G |
| 2 | Anastasis et al | South Africa | Southern Africa | 1997 | Retrospective | Sub national | LJ | 30 | 178 | F |
| 3 | Kenyon et al | Botswana | Southern Africa | 1999 | Cross sectional | Sub national survey | LJ | 52 | 430 | G |
| 4 | Murray et al | South Africa | Southern Africa | 1999 | Retrospective | Sub-national survey | LJ | 6 | 375 | G |
| 5 | Edgbola et al | Gambia | West Africa | 1999 | Cross sectional | National survey. | LJ | 1 | 210 | F |
| 6 | Tudó et al | Equatorial Guinea | Central Africa | 1999 | Cross sectional | Sub national survey | LJ/ radiometric BACTEC | 4 | 224 | F |
| 7 | Urassa et al | Tanzania | East Africa | 2001 | Cross sectional | Sub-national survey.  TB health facilities | LJ | 1 | 280 | F |
| 8 | Mac-Arthur et al | Mozambique | East Africa | 2001 | Cross sectional | Sub-national survey | LJ | 72 | 709 | G |
| 9 | Colette et al | Chad | Central Africa | 2002 | Prospective | Sub national survey | LJ/  MGIT | 0 | 40 | F |
| 10 | Affolabi et al | Benin | West Africa | 2002 | Prospective | Sub-national survey. | LJ | 4 | 244 | G |
| 11 | Nunes et al | Mozambique | East Africa | 2002 | Prospective | Sub-national survey. | LJ | 6 | 38 | F |
| 12 | Bruchfeld et al | Ethiopia | East Africa | 2002 | Prospective | Sub national | LJ | 1 | 121 | F |
| 13 | Nelson et al | Botswana | Southern Africa | 2002 | Cross sectional | National survey. | LJ | 10 | 1990 | G |
| 14 | Asmamaw et al | Ethiopia | East Africa | 2004 | Prospective | Sub-national survey. | LJ | 1 | 173 | F |
| 15 | Ellis Awusu-Dabo et al | Ghana | West Africa | 2004 | Cross sectional | National survey. | LJ | 39 | 2064 | G |

|  | Author | Country | Region | Year of study | Study design | Evaluation | Culture | Positive MDRTB | Number of new Tuberculosis cases | Quality grading |
| --- | --- | --- | --- | --- | --- | --- | --- | --- | --- | --- |
| 16 | Calver et al | South Africa | Southern Africa | 2005 | Retrospective | Sub-national survey | MGIT | 77 | 3003 | G |
| 17 | N'guesesan et al | Ivory Coast | West Africa | 2005 | Cross sectional | National survey. | LJ | 8 | 320 | G |
| 18 | Chonde et al | Tanzania | East Africa | 2006 | Cross sectional | National survey. | LJ | 12 | 1019 | G |
| 19 | Mulenga et al | Zambia | East Africa | 2006 | Prospective | Sub-national survey | LJ | 1 | 361 | G |
| 20 | Matee et al | Tanzania | East Africa | 2006 | Cross sectional | Sub-national survey: | LJ | 3 | 226 | G |
| 21 | Gudo et al | Mozambique | Central Africa | 2007 | Cross sectional | National survey. | LJ | 38 | 1101 | G |
| 22 | Umubyeyi et al | Rwanda | Central Africa | 2007 | Cross sectional | Sub national survey | LJ | 24 | 616 | G |
| 23 | Ramarokoto et al | Madagascar | East Africa | 2007 | Cross sectional | National survey | LJ | 2 | 926 | G |
| 24 | Lawson et al | Nigeria | West Africa | 2007 | Cross sectional | Sub national survey | BACTEC TB | 4 | 32 | F |
| 25 | Asiimwe et al | Uganda | East Africa | 2008 | Prospective | Sub-national survey | LJ | 15 | 344 | G |
| 26 | Yimer et al | Ethiopia | East Africa | 2008 | Cross sectional | Sub-national survey. | MGIT | 1 | 112 | F |
| 27 | Lukoye et al | Uganda | East Africa | 2008 | Cross sectional | Sub-national survey. | LJ | 5 | 473 | G |
| 28 | Sanders et al | Burundi | Central Africa | 2008 | Cross sectional | Sub-national survey. | LJ | 7 | 496 | G |

|  | Author | Country | Region | Year of study | Region | Study design | Evaluation | Culture | Positive MDRTB | Number of new Tuberculosis cases | Quality grading |
| --- | --- | --- | --- | --- | --- | --- | --- | --- | --- | --- | --- |
| 29 | Mbulo et al | Zambia | Central Africa | 2008 | Central Africa | Cross sectional | National survey | LJ | 14 | 883 | G |
| 30 | Bazira et al | Uganda | East Africa | 2008 | East Africa | Cross-sectional | Hospital base ed assessment | LJ/Genotype | 2 | 122 | F |
| 31 | BT Pokam et al | Nigeria | West Africa | 2008 | West Africa | Cross sectional | Sub national survey | LJ | 6 | 97 | F |
| 32 | Mineme-Lingoupou et al | Central African Republic | Central Africa | 2009 | Central Africa | Cross sectional | Sub-national survey. TB health facilities in Bangui and Bimbo. | LJ | 1 | 233 | G |
| 33 | Diande et al | Burkina Faso | West Africa | 2009 | West Africa | Cross sectional | Sub national survey | LJ | 10 | 314 | G |
| 34 | Abdelhadi et al | Chad | Central Africa | 2009 | Central Africa | Cross sectional | Sub-national survey. | LJ | 3 | 135 | F |
| 35 | Tessema et al | Ethiopia | East Africa | 2009 | East Africa | Cross sectional | Sub-national survey. Five | LJ | 13 | 260 | G |
| 36 | Sanchez-Padilla et al | Swaziland | Southern Africa | 2009 | Southern Africa | Cross-sectional | survey | BACTEC MGIT | 27 | 840 | G |
| 37 | Daniel O et al | Nigeria | West Africa | 2010 | West Africa | Retrospective | Sub-national survey | Culture | 3 | 123 | F |
| 38 | Sangare et al | Bukina Furso | West Africa | 2010 | West Africa | Cross sectional | National survey. | LJ | 11 | 323 | G |
| 39 | Cox et al | South Africa | Southern Africa | 2010 | Southern Africa | Cross-sectional | Sub national survey | LJ/LPA | 9 | 271 | G |
| 40 | Ndungu et al | Kenya | East Africa | 2010 | East Africa | Cross sectional | Sub-national survey. Five | MGIT/LJ | 2 | 286 | G |
| 41 | Abebe et al | Ethiopia | East Africa | 2010 | East Africa | Cross sectional | Sub national survey | LJ/BACTEC MGIT | 2 | 156 | F |
| 42 | Abouyannis et al | Malawi | East Africa | 2010 | East Africa | Cross sectional | National survey | LJ | 5 | 1196 | G |

|  | Author | Country | Region | Year of study | Study design | Evaluation | Culture | Positive MDRTB | Number of new Tuberculosis cases | Quality grading |
| --- | --- | --- | --- | --- | --- | --- | --- | --- | --- | --- |
| 43 | Aliyu et al | Nigeria | West Africa | 2010 | Cross sectional | Sub national survey | LJ | 5 | 354 | G |
| 44 | Sangare et al | Bukina Furso | West Africa | 2011 | Cross sectional | Sun national survey | LJ | 2 | 249 | G |
| 45 | Lukoye et al | Uganda | East Africa | 2011 | Prospective | Sub national | LJ | 5 | 557 |  |
| 46 | Irenious et al | Somalia | East Africa | 2011 | Cross sectional | National survey. | Hain | 39 | 754 | G |
| 47 | Halilu et al | Nigeria | West Africa | 2012 | Cross sectional | Sub national survey | Xpert | 22 | 103 | F |
| 48 | South African Tuberculosis Drug Resistance Survey | South Africa | Southern Africa | 2012 | Prospective | National survey | LJ | 22 | 10044 | G |
| 49 | Otu et al | Nigeria | West Africa | 2012 | Cross sectional | Sub national survey | LJ | 4 | 100 | G |
| 50 | Mekonnen et al | Ethiopia | East Africa | 2014 | Cross-sectional | Sub national survey | LJ/GeneXpert MTB/RIF. | 2 | 88 | F |
| 51 | Okorie et al | Nigeria | West Africa | 2015 | Cross sectional | Sub-national. | Xpert MTB/RIF | 13 | 493 | G |
